# Medical and non-medical factors that affect voluntary living-related kidney donation: A single-center study

**DOI:** 10.4103/0971-4065.75223

**Published:** 2011

**Authors:** I. Veerappan, N. Neelakantan, V. Tamilarasi, G. T. John

**Affiliations:** Department of Nephrology, M. S. Ramaiah Memorial Hospital, Bangalore, India; 1 Department of Biostatistics, Christian Medical College, Vellore, Tamil Nadu, India; 2 Department of Nephrology, Christian Medical College, Vellore, Tamil Nadu, India

**Keywords:** Donor selection, living kidney donor, transplantation

## Abstract

The aim was to evaluate the patients with chronic kidney disease stage 5 (CKD 5) and their prospective renal transplant donors with regard to their renal replacement choices, and to assess the medical and non-medical factors that affect living-related renal donor selection. Over 24 months, consecutive patients with CKD 5 and their relatives were interviewed at presentation. Reasons for the choice of modality were analyzed; the prospective recipients and their donors were again interviewed separately and the medical and nonmedical factors that affected the donor selection were determined. A total of 1257 patients were enrolled. Conservative therapy, maintenance dialysis, and renal transplantation were chosen by 513 (40.8%), 320 (25.5%), and 424 (33.7%) patients, respectively. Only socioeconomic status affected the modality chosen. The age, gender, and donor availability did not emerge as significant factors. Patients or donors were likely to withdraw from transplant evaluation due to the absence of a voluntary donor, presence of a male donor, coercion not to donate, and the absence of reimbursement. The commonest cause of rejection of a donor was blood group incompatibility (45.8%), followed by diabetes mellitus (DM) or risk of DM (24%), renal disease (5.9%), hypertension (5.5%), and persistent cross-match positivity (5.1%). To improve donation rates, the donor’s spouse should be involved in the early stages of donor evaluation, financial support for the recipient has to be improved, and the apprehensions about complications of nephrectomy among the donors need to be alleyed. Male donors are at increased risk of leaving the program in the evaluation phase.

## Introduction

Chronic kidney disease is a worldwide public health problem with an increasing incidence and prevalence. An increasing number of patients are treated with renal replacement therapy (RRT) – dialysis or transplantation. The annual incidence of end-stage kidney disease (ESRD) has doubled over the past decade to reach about 135 per million in Europe and a similar rate is seen in USA. It is expected to continue to rise at an annual rate of around 5–8%. In India, the annual incidence is 34–240 per million population.[1–4]

Increasing elderly population and the diabetic epidemic worldwide are largely responsible for the increasing incidence of ESRD. The number of people with diabetes worldwide (currently about 154 million) is predicted to double within the next 20 years.[5] As per the Diabetes Atlas 2006 published by the Diabetes Federation, the number of patients with diabetes in India (currently around 40.9 million) is expected to rise to 69.9 million by 2025 unless urgent preventive measures are taken.[6] This is bound to lead to a parallel epidemic of diabetic nephropathy.

In India, nearly 18,000–20,000 patients (10% of new ESRD cases) get RRT.[7] In 2004, poor Indians spent 40% of their income on health care; the rich spent about 2.4%. Studies have shown that medical expenses were one of the three main factors pushing people into poverty.[8]

The social factors and the perception of the complications of donation by the donor, family members, or even the recipient can affect the act of voluntary donation. In India, the Human Organ Transplantation Act of 1994 and its amendments discourages unrelated transplant due to ethical reasons and to avoid exploitation of the financially disadvantaged people. Data from India on the factors that determine the donor selection are lacking. This study was an effort to explore the medical and nonmedical factors that affect the donor selection.

## Patients and Methods

The study was a prospective study on consecutive patients with chronic kidney disease stage 5 (CKD 5) who presented to the nephrology services of Christian Medical College, Vellore. The renal replacement options chosen by the patients and the medical and nonmedical factors that determine the living kidney donor selection were studied.

Subjects with newly diagnosed CKD 5 from December 2006 to November 2008 were recruited. The diagnosis was based on clinical evaluation, imaging studies including ultrasound, and estimated glomerular filtration rate (GFR) calculated using the abbreviated Modification of Diet in Renal Disease (MDRD) equation. Patients diagnosed as CKD or where chronicity was doubtful prior to the study period were excluded.

The patients and the relatives were interviewed with regard to the planned renal replacement options. The prospective recipients and their prospective donors were interviewed separately. The medical and nonmedical factors that could possibly have a bearing on the selection and rejection or withdrawal of a donor were analyzed.

Descriptive statistics and tests of significance (chi-square tests for categorical variables, Student’s *t*-tests for continuous variables, and logistic regression) were done using the SPSS version 14 (SPSS Inc, Chicago) software package.

## Results

Out of the 1257 CKD 5 patients, 513 (40.8%) had chosen conservative treatment. Poor finances influenced the decision in 466 (96.1%) of the 485 patients otherwise fit for transplantation. No suitable donor was available in 16.

A similar trend was seen among the 320 (25.5%) patients who chose long-term dialysis as RRT. Here 217 (93.5%) of the 232 patients were fit to be a renal transplant recipient, and the decision was influenced by poor finances.

Of the 1257 patients with CKD stage 5, 424 (33.7%) chose renal transplant as their modality of treatment and of these 382 (90.1%) came for recipient evaluation. Seventy-three (19.1%) of the 382 patients did not have any donor and that contributed to 35.3% (73/207) of the prospective recipients who left our transplant program during subsequent evaluation due to various reasons.

**Figure 1:**
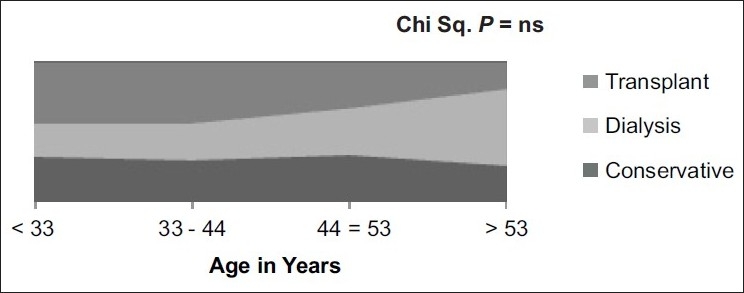
Age and choice of renal replacement therapy

We noted a trend toward older patients choosing maintenance dialysis as a modality of RRT and younger people choosing renal transplantation but it was not statistically significant. In addition, the number of patients choosing conservative treatment was not different across the various age groups suggesting age was not a major factor influencing the choice of therapy [Figure 1].

**Figure 2:**
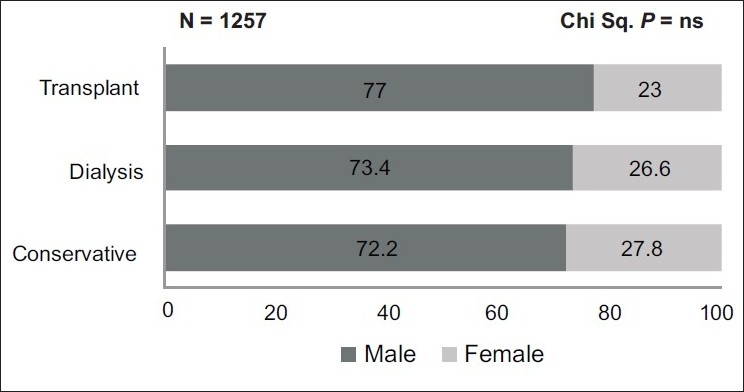
Gender and choice of renal replacement therapy

Similarly, gender had no influence on the choice of therapy and the difference across the groups was not significantly different [Figure 2].

**Figure 3:**
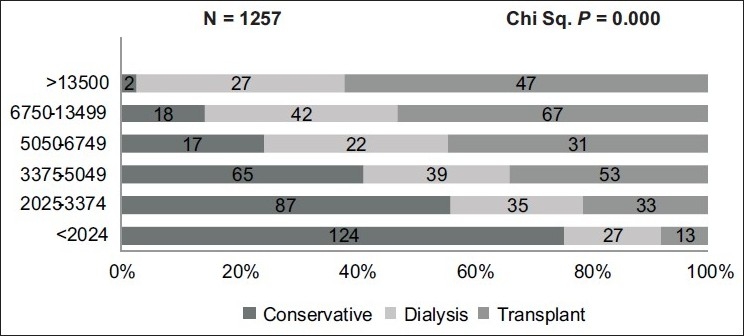
Monthly income and choice of renal replacement therapy

**Figure 4:**
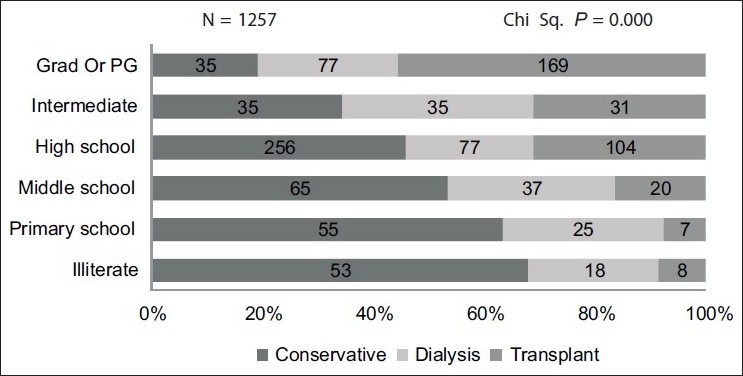
Education and choice of renal replacement therapy

Higher proportion of patient with better education and higher monthly family income chose renal transplant over the other modalities as shown in [Figure 2] and the difference was statistically significant [Figures 3 and 4].

### Demography of the prospective transplant recipients

The mean age of the recipients who came for evaluation was 38.47±13.02 years and the range was from 6 years to 65 years. The recipient population under study was predominantly male forming 74.6% of the cases. The majority of the prospective transplant recipients, i.e., 94.4%, had a level of education better than high school certificate and only one patient was illiterate [Table 1].

**Table 1:** Demography of prospective renal transplant recipients and donors

*N* = 382	Mean±SD or number (%)
Recipient age (years)	38.47±13.02
Male:female	3:1
Socioeconomic status	
Upper	29 (7.6)
Upper middle	253 (66.2)
Middle	94 (24.6)
Upper lower	6 (1.6)
Lower	0 (0)
ABO blood group (recipient, donor)	
O Pos	193 (50.5), 132 (34.6)
A Pos	69 (18.1), 25 (6.5)
B Pos	95 (24.9), 56 (14.7)
AB Pos	19 (5.0), 3 (0.8)
O Neg	4 (1.0), 3 (0.8)
A Neg	1 (0.3), 0 (0)
B Neg	1 (0.3), 2 (0.5)
Donor relationship to recipient	
Mother	81 (21.2)
Brother	75 (19.6)
Sister	63 (16.5)
Spouse	58 (15.2)
Father	41 (10.4)
Son	14 (3.7)
Daughter	1 (0.3)
Others	49 (12.7)
Requested to consider donation	
Recipient	113 (29.6)
Donor came forward with being requested	107 (28)
Recipient’s physician	98 (25.7)
Recipient’s spouse	27 (7.1)
Recipient’s other family member	37 (9.7)
Attempted to influence against donate	46 (12.1)
Donor’s spouse	22 (5.8)
Other relatives	20 (5.3)
Recipient’s physician	4 (1)
Source of funding for transplant	
Self-payment	217 (56.8)
Partly sponsored	69 (18.1)
Fully sponsored	96 (25.1)

As per the modified Kuppusamy classification (urban),[9] 18.1% of the prospective transplant recipients were semiskilled or unskilled workers, 24.6% were semiprofessional or professional, and the rest were skilled workers or were self-employed, or were doing clerical jobs. None of the patients belonged to low socioeconomic status and 90.8% belonged to either upper middle class or middle class [Table 1].

The commonest blood groups in both the recipient and the donor populations were O positive and B positive. The O positive blood group was seen in 50.5% of the recipients while it was seen in 34.6% of the prospective donors. As our program strongly encourages first-degree relatives to donate, most of the donors were near relatives. Mother was the donor in 21.2% of cases and brother, sister, and father were donors in 19.6%, 16.5%, and 10.4%, respectively. Spouse came forward for donation in 68 (15.2%) cases [Table 1].

The donor was first asked by the recipient to donate in 29.6% of cases; a first-degree relative came forward himself/herself in 28% of the cases. The physician helped with donor selection in 25.7% of the cases. In 46 (22%) of cases, there was at least one instance of an attempt to discourage donation and the donor’s spouse was the commonest identified cause in 47.8% of the cases. The commonest reason for a donor not coming forward to donate was spouse’s unwillingness. One case of donor withdrawing consent was due to medical reasons (horseshoe kidney) in the donor. Out of the 382 prospective recipients, 56.8% (217/382) were not eligible for reimbursement by governmental or nongovernmental institutions or by medical insurance [Table 1].

The commonest cause of donor rejection was ABO incompatibility in 45.8% followed by 24% with diabetes or risk of diabetes and renal disease in 5.9% (36/613) in prospective donors. We rejected donors if either of their parents was diabetic or if the status of diabetes was not known in parents and if more than one of the sibling was diabetic. We did consider some of the at-risk group prospective donors who were older and had no impared glucose tolerance. There was a delay (defined arbitarily as duration of more than 30 days) in getting a renal transplant surgery in 91.1% of patients. The medical reasons contributed to 29.6% of the delay. The commonest nonmedical reason was government procedures in 26.4% (101/382) [Table 2].

**Table 2:** Reasons for rejection of a donor

*N =* 613	No. of patients	Percentage
ABO incompatibility	281	45.8
Diabetes mellitus^a^	147	24.0
Renal disease	36	5.9
Proteinuria	17	
Renal calculi	12	
eGFR^b^<60 ml/min	5	
Cortical scar	2	
Proteinuria	17	
Renal calculi	12	
eGFR^b^ <60 ml/min	5	
Cortical scar	2	
Hypertension^c^	34	5.5
Persistent cross-match positive	31	5.1
Liver disease^d^	22	3.6
Ischemic heart disease	17	2.7
Unrelated donor	10	1.6
Pregnancy	4	0.7
Sickle cell anemia/trait	4	0.7
Family h/o ADPKD	4	0.7
Rheumatoid arthritis	2	0.3
Thyroid malignancy	2	0.3
Seizure disorder	2	0.3
SLE	1	0.2
Autoimmune hemolytic anemia	1	0.2
Bronchial asthma (severe persistent)	1	0.2
Mental retardation	1	0.2
Depression	1	0.2
Suspected renal cell carcinoma	1	0.2
RHD severe mitral stenosis	1	0.2

a DM = Diabetes mellitus or family history of diabetes mellitus in either of the parents or more than one sibling if the status of diabetes is not known in the parents

b MDRD eGFR <60 ml/min

c Hypertension = Hypertension before 40 years or requiring more than two drugs at control of blood pressure

d Liver disease = cirrhosis, HbsAg +, HCV antibody +, nonalcoholic fatty liver, alcoholic liver disease

The risk factors we identified among the prospective recipients who left our program were the absence of any donor, presence of male donor, attempted coercion to donate, and absence of financial support by the governmental or nongovernmental institutions either fully or partly [Table 3].

**Table 3:** Characteristics of patients who left versus who got transplantation done/are awaiting

*N* = 382	Transplant done/awaiting	Left	*P* value
Socioeconomic status			ns
Upper	12	18	
Upper middle	109	142	
Middle	48	46	
Upper middle	5	1	
Marital status			ns
Married	122	132	
Unmarried	52	74	
Widow/widower	1	1	
Decline to donate			ns
Nil	149	164	
≥1	26	43	
Rejected			ns
Nil	74	79	
≥1	101	128	
No donor	21	62	0.00
Donor gender			0.03
Male	71	115	
Female	104	92	
Donor marital status			ns
Unmarried	35	59	
Married	129	138	
Divorcee	4	8	
Widow/widower	7	2	
Relationship with the donor			ns
Mother	48	34	
Brother	33	42	
Sister	28	35	
Wife	24	22	
Father	16	25	
Others	82	49	
Requested to consider donation			ns
Recipient	56	57	
Came forward himself/herself	54	54	
Recipient’s physician	45	52	
Recipient’s spouse	8	19	
Recipient’s family other member	12	25	
Attempted to influence decision	17	29	0.013
Perception of complication (unsure or likely)	69	86	ns
Source of finance			
Self	84	132	0.05
Sponsored	91	75	

ns = Not significant

## Discussion

Chronic kidney disease has become an important public health problem. Though life style modification, prevention and early detection of kidney disease prolong life with reasonable quality, the ever-widening gap between the numbers of people waiting for renal transplantation is all too familiar. In our country, our perception of the patient’s choice of the modality of RRT is that it is largely dependent on the means of financial support for transplantation followed by donor factors. We evaluated if that was really the case.

We observed that age did not have a significant influence on the modality chosen. The males constituted 77.2% of the total population. This was comparable to the 69.6% as per the Indian CKD registry.[10] Though it is tempting to believe that more males had sought medical attention may be due to socioeconomic factors, it is likely that the incidence of the disease *per se* is more in males. In a study in a French urban area, the male:female ratio was 2:1 after the age of 20 years onward.[11] Hospital-based data from south India showed a prevalence of 60–70% of males with CKD.[2] However, in an urban community-based study from India, only 48% of the population with any stage of CKD was males.[3]

There was no significant difference in the male:female ratio in the three modalities of therapy chosen by the patient suggesting that age and gender were not the major factors involved in choosing a particular modality of RRT. Even in countries where the treatment is state sponsored, males outnumber females. Some of the gender difference in prevalence may be due to risk factors shared by cardiovascular disease and end-stage renal failure, both of which are commoner in men.

There was a significant difference in the monthly family income, education status, and socioeconomic status of the three treatment groups with higher income group and patients with better education opting for transplant and dialysis compared to the group that chose conservative treatment. In our study, only 7.6% were fully sponsored and 5.5% were partly sponsored by governmental or nongovernmental organizations for the treatment expenses. In 2004, poor Indians spent 40% of their income on health care; the rich spent about 2.4%.[8] Studies have shown that medical expenses were one of the three main factors pushing people into poverty. Hence it is not surprising that a large proportion of patients chose conservative treatment.[8]

We did not evaluated the “health-related literacy” as a part of our study with instruments like Short Test of Functional Health Literacy in Adults (STOHFLA) which would have affected the decision-making process. However, the majority of the prospective transplant recipients, i.e., 94.4%, had a level of education better than high school certificate and only one patient was illiterate.

As per the modified Kuppusamy classification (urban),[9] 18.1% of the prospective transplant recipients were semiskilled or unskilled workers. None of the patients belonged to low socioeconomic status and 90.8% were either from upper middle class or middle class. Hence the only way of improving the transplantation rates in the country is to support these patients financially.

The number of patients who chose conservative treatment, dialytic treatment, and renal transplant were 40.8%, 25.5%, and 33.7%, respectively, as against the Indian CKD registry data of 75.1%, 22.5%, and 2.5%, respectively.[10] Most of the patients who started dialysis left our center and it is possible that a large proportion of them stopped their planned long-term dialysis due to financial reasons. In an earlier study done in the same center in 1993, only 3.6% of the 463 patients remained on chronic dialysis 4 weeks after initiation and the median follow-up duration in that study was 16 months.[12] Of the 33.7% of the patients who had chosen renal transplant as their modality of treatment, 54.2% had left for various reasons leaving only 13.9% of the initial CKD 5 population with us for further evaluation. Such a large number of patients choosing renal transplantation as a modality can be due to two reasons. The first is the fact that only 17.7% were from the neighboring districts of Tamil Nadu, Andhra Pradesh, and Kerala and a significant number traveled over 1500–2000 km to our center for treatment, and only people with enough means can do so. Some of the patients who left our center possibly received treatment at another center offering more affordable treatment. The second reason is that our center is a referral hospital and a significant number of patients are referred only for renal transplantation. So our findings are not representative of the general population.

For the 172 patients who either had a transplant or were in the final stages of evaluation, 70.9% were males. Females were the donors for 59.8% of the recipients. Muthusethupathi *et al*. from a state-funded hospital from the state of Tamil Nadu, India, had reported that two-thirds of their donor population was females and this had only marginally changed over the last 10 years.[13] Interestingly, for the 1725 possible age compatible donors for all the 382 prospective recipients in our study, the male:female ratio was 1:1. This gender difference is possibly due to increased prevalence of renal disease in males, gender differences in access to medical care, and psychosocial and cultural factors, and differences in the perceived risk of transplantation, assertiveness, motivation, attitudes, beliefs, and economic loss with donation.[14,15]

The donors volunteered themselves for transplant evaluation in 28% of cases and in the rest, it was requested by the recipient or suggested by the recipient’s physician. Of all the donors who came for evaluation, 46 (22%) reported at least one instance of an attempt to discourage donation and donor’s spouse was the commonest (47.8%) identified cause. Similarly, the commonest cause for some of the possible donors declining to even come forward for donor evaluation was again the spouse refusing to give consent. And in the rest, no reason was disclosed. This emphasizes the fact that the donor’s spouse must be a part of decision making along with the donor to alley some of the fears associated with donation. In fact, despite the donors voluntarily donating the organ, 40.5% were either unsure or felt it was likely (38.7%) to have complications peroperative or have long-term morbidity like renal failure (1.8%). Imparting better education of the immediate and long-term complications with kidney donation to the general public and the prospective donors will for sure alley some of the fear about the surgery and its complications and may increase the donation rates.

**Figure 5:**
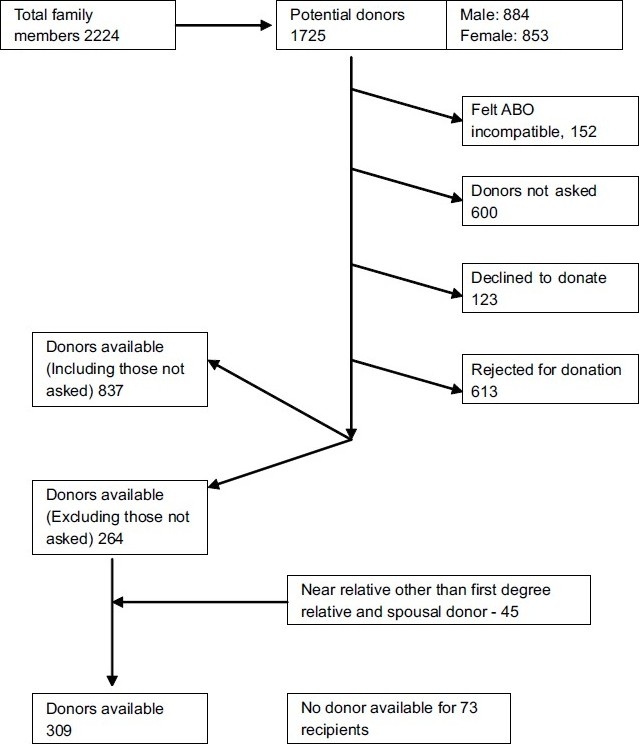
Algorithm showing the factors involved in donor selection

For the 382 prospective recipients, there were 1725 age compatible donors with 4.5 donors per recipient. Interestingly the male female ratio was approximately 1:1 among the total number of the donors [Figure 5]. One-third of the donor pool was never requested for donation, 8.8% was ABO blood group incompatible, 7.1% declined to donate, and 35.5% were rejected from donation upon evaluation, based on clinical investigations. Despite 45 near relatives other than the first-degree relative and spousal donors who consented to donate, 19.1% prospective recipients did not have a donor.

We observed that compared to the group that successfully got a transplant or was waiting for the transplant surgery, the group of patients who left our center without renal transplant had more number of prospective recipients who did not have a donor (30%), had less female prospective donors (44.4%), their donors were more likely to be coerced not to donate (14%), and were less often sponsored (36.2%).

## Conclusion

To improve donation rates, the donor’s spouse should be taken into confidence in the early stages of donor evaluation; financial support for the recipient have to be improved; and the apprehensions about complications of the renal transplant surgery among the donors to be alleyed both for individual donors and the general public. The prospective recipients with male donors are at increased risk of leaving the program in the evaluation phase.
